# 2D-DOD and 2D-DOA Estimation for a Mixture of Circular and Strictly Noncircular Sources Based on L-Shaped MIMO Radar

**DOI:** 10.3390/s20082177

**Published:** 2020-04-12

**Authors:** Jiaxiong Fang, Yonghong Liu, Yifang Jiang, Yang Lu, Zehao Zhang, Hua Chen, Laihua Wang

**Affiliations:** 1 School of Faculty of Information Science and Engineering, Ningbo University, Ningbo 315211, China; a1024758276@163.com (J.F.); liuyonghong4627@126.com (Y.L.); koyo4458@163.com (Y.J.); ly2691413661@163.com (Y.L.); zzhnightbacker@163.com (Z.Z.); 2 Key Laboratory of Intelligent Perception and Advanced Control of State Ethnic Affairs Commission, Dalian 116600, China; 3 School of Software, Qufu Normal University, Qufu 273165, China; wlh@tju.edu.cn

**Keywords:** MIMO radar, four dimensional (4D) angle estimation, noncircular signal, joint diagonalization, stochastic Cramer–Rao bound (CRB)

## Abstract

In this paper, a joint diagonalization based two dimensional (2D) direction of departure (DOD) and 2D direction of arrival (DOA) estimation method for a mixture of circular and strictly noncircular (NC) sources is proposed based on an L-shaped bistatic multiple input multiple output (MIMO) radar. By making full use of the L-shaped MIMO array structure to obtain an extended virtual array at the receive array, we first combine the received data vector and its conjugated counterpart to construct a new data vector, and then an estimating signal parameter via rotational invariance techniques (ESPRIT)-like method is adopted to estimate the DODs and DOAs by joint diagonalization of the NC-based direction matrices, which can automatically pair the four dimensional (4D) angle parameters and solve the angle ambiguity problem with common one-dimensional (1D) DODs and DOAs. In addition, the asymptotic performance of the proposed algorithm is analyzed and the closed-form stochastic Cramer–Rao bound (CRB) expression is derived. As demonstrated by simulation results, the proposed algorithm has outperformed the existing one, with a result close to the theoretical benchmark.

## 1. Introduction

A multiple input multiple output (MIMO) radar can provide increased degrees of freedom by exploiting waveform diversity, with an enhanced performance for spatial resolution, parameter estimation, and target detection [1,2,3,4,5,6,7]. In MIMO radar, by focusing on both directions of departure (DODs) and directions of arrival (DOAs), target localization [8,9,10] is an important issue that has drawn significant attention in recent years.

For MIMO radar systems based on one dimensional (1D) uniform linear arrays (ULAs), in reference [11], by employing the property of Kronecker product, a reduced-dimension multiple signal classification (MUSIC) method was developed, and only 1D search was required to locate the DOD and DOA of the target. A double polynomial root MUSIC method was proposed to jointly estimate the DOA and DOD in [12]. To avoid an exhaustive search over the whole angle space, joint DOA and DOD estimation methods were proposed based on the computationally efficient estimating signal parameter via rotational invariance techniques (ESPRIT) method with pairing required in reference [13] and pairing-free in reference [14], respectively. Furthermore, joint diagonalization-based ESPRIT method was presented in reference [15], where a closed-form expression for both DOA and DOD was obtained and automatically paired. For MIMO radar systems based on planar arrays, a method for joint estimation of 2D-DOD and 2D-DOA was presented in references [16,17,18] by transforming the four dimensional (4D) angle estimates into four 1D estimates without any pairing procedures. In addition, the method in reference [17] can also work well when common 1D angles are presented by joint diagonalization, but the method in reference [16] cannot. Based on the electromagnetic vector sensors (EVSs), 2D-DODs and 2D-DOAs were estimated using an ESPRIT-based method for the bistatic MIMO radar, with an additional optimization function for pair matching [18]. 

However, the above-mentioned methods for MIMO radar failed to exploit some potential information of radar signals, such as noncircularity [19,20,21,22,23], which could lead to significant improvement in its performance. With the aid of noncircular (NC) property of the signals, a series of angle estimation methods [24,25,26] for bistatic MIMO radar were proposed, which can improve the accuracy of angle estimation and detect more signals. In reference [24], with NC incoming signals, a combined ESPRIT and MUSIC approach was applied to MIMO radar for joint estimation of DOA and DOD by decoupling the 2D direction findings into two 1D ones. In reference [25], a kind of ESPRIT algorithm of low complexity was proposed with real-valued computation by Euler’s formula, for MIMO radar with NC signals employed, but it requires additional cost function to avoid ambiguity. For a more general situation with the coexistence of noncircular and circular signals, ESPRIT and unitary ESPRIT were employed in reference [26] for MIMO radar; however, no theoretical error performance analysis was provided for the proposed method, and the derived stochastic Cramer–Rao bound (CRB) does not have a closed-form expression.

In this paper, a NC ESPRIT-like method based on joint diagonalization is proposed to estimate 2D-DOD and 2D-DOA for an L-shaped bistatic MIMO radar. The main contributions of the work are given as follows.

(1) A general model including a mixture of circular and strictly noncircular sources is built for the L-shaped bistatic MIMO radar by stacking received data vector and its conjugated counterpart. Four NC-based direction matrices are then constructed and by joint diagonalization an ESPRIT-like algorithm is developed employing four block selection matrices.
(2) The proposed algorithm can work in the case of common 1D DODs and DOAs, and automatically pair the 4D angle parameters.
(3) The asymptotic performance of the proposed algorithm is analyzed, and the stochastic Cramer–Rao bound (CRB) for the problem is derived with a closed-form expression to serve as the performance benchmark.

The rest of this paper is organized as follows. Section 2 introduces the general mixed signal model for MIMO radar. The proposed algorithm is described in detail in Section 3. The asymptotic performance of the proposed algorithm and the closed-form stochastic CRB are analyzed in Section 4. Simulation results are presented in Section 5, and conclusions are drawn in Section 6.

Notations: ${\left(\cdot \right)}^{*}$, ${\left(\cdot \right)}^{T}$, ${\left(\cdot \right)}^{-1}$, and ${\left(\cdot \right)}^{H}$ denote conjugate, transpose, inverse, and conjugate transpose, respectively. E(⋅) and var(⋅) are the expectation and variance operations, respectively; Re(⋅) and Im(⋅) denote the real and imaginary parts; diag(⋅) denotes the diagonal matrix; blkdiag(⋅) represents the generation of a block diagonal matrix; ⊗ and ⊙ are the Kronecker and Hadamard products, respectively; $I_{k}$ denotes the k-dimensional identity matrix; $\gamma_{k}$ represents the k-dimensional exchange matrix; $0_{k\times l}$ and $1_{k\times l}$ denote the k×l zero matrix and all-one matrix, respectively; arg(⋅) is the phase operation; and tr(⋅) represents the trace of a matrix.

## 2. General Signal Model

**Figure 1:**
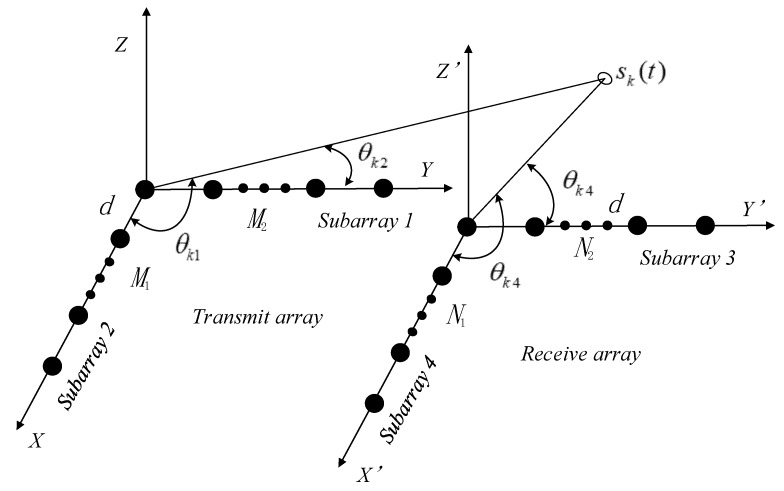
A diagram for the L-shaped multiple input multiple output (MIMO) array structure.

Consider a bistatic MIMO radar system with an L-shaped antenna array for signal transmission and a second L-shaped antenna array for signal reception, as shown in Figure 1. It is assumed that the target fluctuates according to the Swerling II model [1,2,3,4], i.e., the reflection coefficient changes from pulse to pulse. The transmit array has a total number of $M=M_{1}+M_{2}-1$ antennas, with $M_{1}$ and $M_{2}$ antennas located on the X and Y axes, respectively, and the receive array has $N=N_{1}+N_{2}-1$ antennas, of which $N_{1}$ and $N_{2}$ elements are located on the $X^{\prime }$ and $Y^{\prime }$ axes, respectively. The four subarrays are all uniform linear arrays (ULAs) with omnidirectional antennas and a half-wavelength inter-element spacing d. The M transmitted waveforms are supposed to be circular (QPSK)- or strictly noncircular (BPSK)-modulated. The targets that slowly move are far field with their directions parameterized as $\left(\theta_{k1},\theta_{k2},\theta_{k3},\theta_{k4}\right)$, where $\left(\theta_{k1},\theta_{k2}\right)$ is the 2D-DOD of the kth target and $\left(\theta_{k3},\theta_{k4}\right)$ is its 2D-DOA. The received signals reflected by *K* targets at the receive array can be written as

(1) $$r(l,t)=\sum_{k=1}^{K}\alpha_{k}(t)a(\theta_{k1},\theta_{k2})b^{T}(\theta_{k3},\theta_{k4})u(l,t)+w(l,t)$$

where $a_{k}=a(\theta_{k1},\theta_{k2})$ and $b_{k}=b(\theta_{k3},\theta_{k4})$ are the M×1 transmit array and N×1 receive array manifold vectors, with $a_{k}={\left[e^{j\eta dM_{2}\cos\theta_{k2}},\cdots ,1,\cdots ,e^{j\eta dM_{1}\cos\theta_{k1}}\right]}^{T}$ and $b_{k}={\left[e^{j\eta dN_{2}\cos\theta_{k4}},\cdots ,1,\cdots ,e^{j\eta dN_{1}\cos\theta_{k3}}\right]}^{T}$, $\eta =2\pi \lambda^{-1}$, and $u(\tau ,t)={\left[u_{1}^{T}(\tau ,t),\cdots ,u_{M}^{T}(\tau ,t)\right]}^{T}$; $u_{m}(\tau ,t)$ is the *m*th transmitter antenna signal that is supposed to be circular (QPSK)- or strictly noncircular (BPSK)-modulated, $\alpha_{k}(t)$ is the reflection coefficient of the *k*th target depending on the target radar cross section (RCS), and w(τ,t) is the additive white Gaussian noise vector with zero mean and variance $\sigma_{n}^{2}$. τ and t indicate the time within pulse (fast time) and the index of radar pulse (slow time), respectively. Thus, the output of the matched filters at the receive array can be expressed as

(2) $$\begin{array}{cc} y_{m}(t) & =\sum_{k=1}^{K}a_{k}b_{k}^{T}\alpha_{k}(t)r_{k}(t)\left[\begin{array}{c} 0\\ \vdots \\ 0\\ 1\\ 0\\ \vdots \\ 0 \end{array}\right]+w_{m}(t)\\ & =\sum_{k=1}^{K}a_{k}b_{k,m}\alpha_{k}(t)r_{k}(t)+w_{m}(t)\\ & =\sum_{k=1}^{K}a_{k}b_{k,m}s_{k}(t)+w_{m}(t) \end{array}$$

where $b_{k,m}$ denotes the mth element of the transmitter steering vector, $s_{k}(t)=\alpha_{k}(t)r_{k}(t)$ is circular or strictly noncircular baseband signal, and $w_{m}(t)$ is the noise vector after matched filter. As for strictly noncircular baseband signal, the $s_{k}(t)$ can also be written as $s_{k}(t)=s_{n,k}(t)e^{j\varphi_{k}/2}$ [19,20,21,22,23], where $s_{n,k}(t)$ is real-value and $\varphi_{k}/2$ is arbitrary phase shifts that can be different for each signal but are constant with time.

Let $x(t)=[y_{1}^{T}(t),\ldots y_{M}^{T}(t){]}^{T}$ be the output of all the received signal, which is shown as

(3) $$x(t)=C(\theta_{k1},\theta_{k2},\theta_{k3},\theta_{k4})s(t)+n(t)$$

where x(t) is the MN×1 data vector, $C={\left[c_{1},c_{2},\cdots ,c_{K}\right]}^{T}$ is the MN×K extended virtual array manifold matrix, and $c_{k}=b_{k}⊗a_{k}$ is the MN×1 extended virtual array manifold vector. $n(t)={\left[n_{1}(t),\cdots ,n_{MN}(t)\right]}^{T}$ is the MN×1 additive white Gaussian noise vector with zero mean and variance $\sigma_{n}^{2}$. $s(t)={\left[s_{1}(t),\cdots ,s_{K}(t)\right]}^{T}$ is the K×1 mixed signal vector, which contains $K_{n}$ strictly noncircular signals $s_{n,k}(t),k=1,2,\cdots ,K_{n}$ and $K_{c}$ circular signals $s_{c,k}(t),k=1,2,\cdots ,K_{c}$, satisfying $K=K_{n}+K_{c}$. As shown in [27,28], each of the circular signals can be separated into two uncorrelated strictly noncircular signals. Thus, s(t) can be rewritten as

(4) $$s(t)=\left[\begin{array}{cc} \Phi_{1} & 0\\ 0 & \left[\begin{array}{cc} I_{K_{c}} & jI_{K_{c}} \end{array}\right] \end{array}\right]\left[\begin{array}{c} s_{n}(t)\\ s_{c}^{r}(t)\\ s_{c}^{q}(t) \end{array}\right]=\Phi \tilde{s}(t)\;$$

where $\Phi_{1}=diag\left(e^{j\varphi_{1}/2},\cdots ,e^{j\varphi_{K_{n}}/2}\right)$ is the $K_{n}\times K_{n}$ arbitrary phase matrix corresponding to the strictly noncircular signals $s_{n}(t)$; furthermore, Φ is of size $K\times K^{\prime }$ with $K^{\prime }=K_{n}+2K_{c}$, and the $K^{\prime }\times 1$ real-valued vector $\tilde{s}(t)$ contains the symbols of the $K_{n}$ strictly noncircular signals $s_{n}(t)$ cum the $K_{c}$ real parts $s_{c}^{r}(t)$ and $K_{c}$ imaginary parts $s_{c}^{q}(t)$ of the circular signals $s_{c}(t)$. Therefore, the extended virtual array manifold matrix C can be rewritten as

(5) $$C=\left[\begin{array}{cc} C_{n} & C_{c} \end{array}\right]$$

where $C_{n}$ and $C_{c}$ represent the $MN\times K_{n}$ and $MN\times K_{c}$ array manifold matrix related to strictly noncircular and circular signals, respectively.

According to Equations (4) and (5), the data vector of Equation (3) can be expressed as

(6) $$\begin{array}{cc} x(t) & =C\Phi \tilde{s}(t)\;+n(t)\\ & =C_{n}\Phi_{1}s_{n}(t)+C_{c}s_{c}(t)+n(t) \end{array}$$

For notional convenience, the angle pair $\left(\theta_{k1},\theta_{k2},\theta_{k3},\theta_{k4}\right)$ and time t will be omitted in the following sections.

## 3. The Proposed Algorithm

In order to utilize the noncircularity characteristic of the strictly noncircular signals and the virtual noncircularity characteristic of the circular signals, a new data matrix is constructed by stacking the original data matrix $X=[x(1),\cdots ,x_{MN}(T)]$ (T is the number of snapshots) and its corresponding conjugated counterpart as

(7) $$Y=\left[\begin{array}{c} X\\ \gamma_{MN}X^{*} \end{array}\right]=\left[\begin{array}{c} C\Phi \tilde{S}\\ \gamma_{MN}C^{*}\Phi^{*}{\tilde{S}}^{*} \end{array}\right]+\left[\begin{array}{c} N\\ \gamma_{MN}N \end{array}\right]=\tilde{C}\tilde{S}+\tilde{N}$$

where

(8) $$\tilde{C}=\left[\begin{array}{c} C\Phi \\ \gamma_{MN}C\Phi \end{array}\right]=\left[\begin{array}{cc} C_{n}\Phi_{1} & C_{c}\left[\begin{array}{cc} I_{K_{c}} & jI_{K_{c}} \end{array}\right]\\ \gamma_{MN}C_{n}^{*}\Phi_{1}^{*} & \gamma_{MN}C_{c}^{*}\left[\begin{array}{cc} I_{K_{c}} & -jI_{K_{c}} \end{array}\right] \end{array}\right]$$

is the $2MN\times K^{\prime }$ extended array manifold matrix, $\tilde{N}=\left[\begin{array}{c} N\\ \gamma_{MN}N \end{array}\right]$ is the 2MN×T noise matrix with $N=[n(1),\cdots ,n_{MN}(T)]$, and $\tilde{S}={\tilde{S}}^{*}$ with $\tilde{S}=[\tilde{s}(1),\cdots ,{\tilde{s}}_{MN}(T)]$.

Performing singular value decomposition (SVD) on Y, one can get

(9) $$Y=\left(\begin{array}{cc} U_{s} & U_{n} \end{array}\right)\left(\begin{array}{cc} \Sigma_{s} & 0\\ 0 & \Sigma_{n} \end{array}\right)\left(\begin{array}{c} V_{s}^{H}\\ V_{n}^{H} \end{array}\right)=U_{s}\Sigma_{s}V_{s}^{H}+U_{n}\Sigma_{n}V_{n}^{H}$$

where the $2MN\times K^{\prime }$ matrix $U_{s}$ and the $T\times K^{\prime }$ matrix $V_{s}$ are the left and right singular signal subspace associated with corresponding left and right singular values matrices $\Sigma_{s}=diag\left(\lambda_{1},\lambda_{2},\cdots ,\lambda_{K^{\prime }}\right)$ and $\Sigma_{n}=diag\left(\lambda_{K^{\prime }+1},\lambda_{K^{\prime }+2},\cdots ,\lambda_{2MN}\right)$, respectively, while the $2MN\times \left(2MN-K^{\prime }\right)$ matrix $U_{n}$ and the $T\times \left(2MN-K^{\prime }\right)$ matrix $V_{n}$ are the left and right singular noise subspace, respectively. 

**Figure 2:**
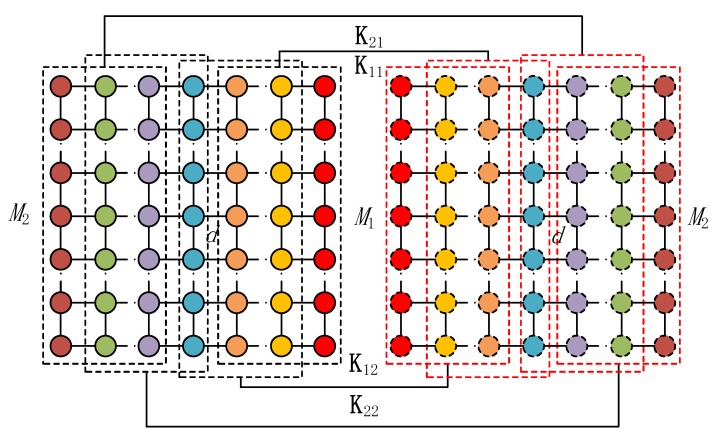
Block selection matrices for estimating $\left(\theta_{k1},\theta_{k2}\right)$.

**Figure 3:**
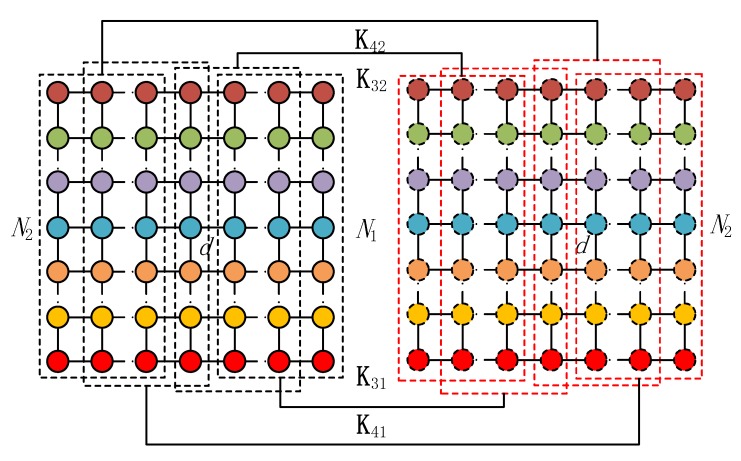
Block selection matrices for estimating $\left(\theta_{k3},\theta_{k4}\right)$.

By defining a new matrix $E_{s}$ as $E_{s}=U_{s}\Sigma_{s}$, and the following selection matrices

(10) $$J_{1a}=\left[\begin{array}{ccc} 0_{\left(a-1\right)\times \left(M-a\right)} & 0_{\left(a-1\right)\times 1} & I_{\left(a-1\right)} \end{array}\right],a=M_{1},N_{1}$$

(11) $$J_{2a}=\left[\begin{array}{ccc} 0_{\left(a-1\right)\times \left(M-a\right)} & I_{\left(a-1\right)} & 0_{\left(a-1\right)\times 1} \end{array}\right],a=M_{1},N_{1}$$

(12) $$J_{1b}=\left[\begin{array}{ccc} I_{\left(b-1\right)} & 0_{\left(b-1\right)\times 1} & 0_{\left(b-1\right)\times \left(M-b\right)} \end{array}\right],b=M_{2},N_{2}$$

(13) $$J_{2b}=\left[\begin{array}{ccc} 0_{\left(b-1\right)\times 1} & I_{\left(b-1\right)} & 0_{\left(b-1\right)\times \left(M-b\right)} \end{array}\right],b=M_{2},N_{2}$$

the selection matrices displayed in Figure 2 for $\theta_{kl}\left(l=1,2\right)$ of the mixed strictly noncircular and circular signals can be expressed as

(14) $$K_{l1}=blkdiag\left(J_{l1},\gamma_{\left(M_{l}-1\right)M}J_{l2}\gamma_{MN}\right),l=1,2$$

(15) $$K_{l2}=blkdiag\left(J_{l2},\gamma_{\left(M_{l}-1\right)M}J_{l1}\gamma_{MN}\right),l=1,2$$

where $J_{11}=I_{N}⊗J_{1M_{1}}$, $J_{12}=I_{N}⊗J_{2M_{1}}$, $J_{21}=I_{N}⊗J_{1M_{2}}$, and $J_{22}=I_{N}⊗J_{2M_{2}}$. Similarly, as shown in Figure 3, the selection matrices for $\theta_{kl}\left(l=3,4\right)$ of the mixed signals can be expressed as

(16) $$K_{l1}=blkdiag\left(J_{l1},\gamma_{\left(N_{l}-1\right)N}J_{l2}\gamma_{NM}\right),l=3,4$$

(17) $$K_{l2}=blkdiag\left(J_{l2},\gamma_{\left(N_{l}-1\right)N}J_{l1}\gamma_{NM}\right),l=3,4$$

where $J_{31}=J_{1N_{1}}⊗I_{M}$, $J_{32}=J_{2N_{1}}⊗I_{M}$, $J_{41}=J_{1N_{2}}⊗I_{M}$, and $J_{42}=J_{2N_{2}}⊗I_{M}$.

Instead of complex peak-seeking methods [29,30,31,32], following the principle of the ESPRIT algorithm [33,34,35], we define the direction matrices $G_{l}$ related to $\theta_{kl}\left(l=1,2,3,4\right)$ as follows

(18) $$G_{l}={\left(K_{l2}E_{s}\right)}^{+}K_{l1}E_{s}=E\Theta_{l}E^{H},l=1,2,3,4$$

where

(19) $$\begin{array}{cc} \Theta_{l} & =diag\left(e^{j2\pi \lambda^{-1}d\cos\theta_{1l}},\cdots ,e^{j2\pi \lambda^{-1}d\cos\theta_{K’l}}\right)\\ & =diag\left(\eta_{1l},\cdots ,\eta_{K’l}\right) \end{array}$$

is a diagonal matrix and E is the $K^{\prime }\times K^{\prime }$ unitary matrix.

It can be seen that $G_{l}$ in Equation (18) satisfies the joint diagonalization condition. Then, we define a set $G=\left\{G_{1},G_{2},G_{3},G_{4}\right\}$ and use the joint diagonalization method in [28,36,37] to obtain the unitary matrix $E=\left[e_{1},e_{2},\cdots ,e_{K^{\prime }}\right]$, where $e_{k}$ is the eigenvector of G. It should be mentioned that the proposed method does not require the 4D angle pairing process, as the eigenvalues of G maintain a one-to-one correspondence in the joint diagonalization process. Then, the eigenvalues of G can be computed as

(20) $$\eta_{kl}=e_{k}^{H}G_{l}e_{k},k=1,2,\cdots ,K^{\prime },l=1,2,3,4$$

Thus, it can be easily obtained that

(21) $$\theta_{kl}=\arccos\left(\frac{\lambda \arg\left(\eta_{kl}\right)}{2\pi d}\right),k=1,2,\cdots ,K^{\prime },l=1,2,3,4$$

It should be noted that each circular signal is treated as two strictly noncircular signals, and $K^{\prime }$ angle estimates are obtained for the mixed targets. However, only K actual 2D-DODs and 2D-DOAs are present, so the circular and strictly noncircular signals can be discriminated according to the number of repetitions of the angle estimates. Then, the two estimated angles of the circular signal are reliable, and $\theta_{kl,c}\left(l=1,2,3,4\right)$ can be obtained by calculating the average of two identical estimates

(22) $$\theta_{kl,c}=\frac{\theta_{kl,c}^{1}+\theta_{kl,c}^{2}}{2},k=1,\cdots ,K_{c}$$

Till now, the proposed method has provided closed-form of 2D-DOA and 2D-DOD angle estimates that are automatically paired and summarized in Table 1.

**Remark 1.** The major computational effort the proposed algorithm contains SVD of $\hat{Y}$, pseudo inverse operation for $G_{l}$ in Equation (18), and joint diagonalization of the set $\hat{G}$. Performing SVD of $\hat{Y}$ requires the amount of complex multiplications of $O\left({\left(2MN\right)}^{2}L\right)$, the pseudo inverse in Equation (18) costs $O\left(4{\left(2MN\right)}^{3}\right)$, and jointly diagonalizing the set $\hat{G}$ is of $O\left(4{\left(K^{\prime }\right)}^{3}\right)$. The total computational complexity of the proposed algorithm is about $O\left({\left(2MN\right)}^{2}L+4{\left(2MN\right)}^{3}+4{\left(K^{\prime }\right)}^{3}\right)$.

**Remark 2.** Compared to the Xia’s algorithm [17], the proposed algorithm exploits the redundancy existing in the noncircular signals, which improves the array virtual aperture. Additionally, the maximum numbers of detectable signals by the proposed algorithm is based on the new data vetor in Equation (7) as well as the matrices $K_{l1}$ and $K_{l2},l=1,2$ in Equations (14) and (15) for 2D DODs, $K_{l1}$ and $K_{l2},l=3,4$ in Equations (16) and Equation (17) for 2D DOAs, which are shown in Table 2 compared to Xia’s algorithm. Obviously, the proposed algorithm can distinguish more mixed signals than Xia’s algorithm.

## 4. Performance Analysis

### 4.1. Asymptotic Performance Analysis

In this section, the asymptotic performance of the proposed algorithm is derived, which is consistent with the first-order analysis done by Rao [38] and the backward error analysis of Li [39]. For the ESPRIT-like subspace algorithm, we need to analyze subspace perturbation as a criterion for evaluation. Therefore, we can perform SVD on the noiseless extended observation model $\tilde{Y}$ as follows:

(23) $$\tilde{Y}=\left(\begin{array}{cc} U_{s^{\prime }} & U_{n^{\prime }} \end{array}\right)\left(\begin{array}{cc} \Sigma_{s^{\prime }} & 0\\ 0 & \Sigma_{n^{\prime }} \end{array}\right)\left(\begin{array}{c} V_{s^{\prime }}^{H}\\ V_{n^{\prime }}^{H} \end{array}\right)$$

In line with the first-order approximation principle [38,39] of eigenvalues in Equation (18), we get

(24) $$\begin{array}{cc} \delta \eta_{kl} & \approx e_{k}^{H}\delta G_{l}e_{k},k=1,2,\cdots ,K^{\prime },l=1,2,3,4\\ & =e_{k}^{H}{\left(K_{l2}U_{y}\right)}^{+}(K_{l1}-\eta_{kl}K_{l2})\delta U_{y}e_{k} \end{array}$$

where

(25) $$U_{y}=U_{s^{\prime }}\Sigma_{s^{\prime }}$$

(26) $$\delta U_{y}=\delta U_{s^{\prime }}\Sigma_{s^{\prime }}$$

(27) $$\delta U_{s^{\prime }}=U_{n^{\prime }}U_{n^{\prime }}^{H}\tilde{N}V_{s^{\prime }}\Sigma_{s^{\prime }}^{-1}$$

According to Equations (26) and (27), Equation (24) can be rewritten as

(28) $$\delta \eta_{kl}\approx e_{k}^{H}{\left(K_{l2}U_{y}\right)}^{+}(K_{l1}U_{n^{\prime }}-\eta_{kl}K_{l2}U_{n^{\prime }})U_{n^{\prime }}^{H}\tilde{N}V_{s^{\prime }}e_{k}$$

By performing the first-order Taylor series expansion on Equation (21), the perturbation of $\theta_{kl}$ can be expressed as

(29) δθkl=ξklIm(δηklηkl)

where $\xi_{kl}=\lambda {\left(2\pi d\sin\theta_{kl}\right)}^{-1}$. The error-variances of the estimated 2D-DODs and 2D-DOAs of the mixed sources are

(30) var(δθkl)=ξkl2var{Im(δηklηkl)}

It is worth noting here that Equation (30) can only calculate the mean-squared error for the strictly noncircular signals, while the variances of the four estimated angles for the kth $k=1,2,\cdots ,K_{c}$ circular signal are calculated as

(31) $$\begin{array}{cc} \mathrm{var}(\delta \theta_{kl,c}) & =\mathrm{var}\left(\frac{\delta \theta_{kl,c}^{1}+\delta \theta_{kl,c}^{2}}{2}\right)\\ & =\frac{\mathrm{var}(\delta \theta_{kl,c}^{1})+\mathrm{var}(\delta \theta_{kl,c}^{2})+2E(\delta \theta_{kl,c}^{1}\delta \theta_{kl,c}^{2})}{4} \end{array}$$

### 4.2. Stochastic Cramer–Rao Bound

The CRB, which has a lower bound on the variance of any unbiased estimator, is often adopted for the performance benchmark. In reference [20], the CRB is analyzed by assuming that the set of incident sources are all strictly noncircular, while the CRB analyzed in reference [40], provided that the incident sources are all circular. However, when considering a scenario that both the strictly noncircular and circular sources coexist, the two signal models mentioned above are not applicable. In this section, the stochastic closed-form CRB is first derived for the estimates of 2D-DOA and 2D-DOA of the mixed strictly noncircular and circular sources based on the L-shaped bistatic MIMO radar. 

Here, we focus on a real-valued vector of the interest parameters $\omega ={\left[\theta_{n1}^{T},\theta_{n2}^{T},\theta_{n3}^{T},\theta_{n4}^{T},\psi_{n}^{T},\theta_{c1}^{T},\theta_{c2}^{T},\theta_{c3}^{T},\theta_{c4}^{T}\right]}^{T}$ with $\theta_{n1}={\left[\theta_{n1,1},\cdots ,\theta_{n1,K_{n}}\right]}^{T}$, $\theta_{n2}={\left[\theta_{n2,1},\cdots ,\theta_{n2,K_{n}}\right]}^{T}$, $\theta_{n3}={\left[\theta_{n3,1},\cdots ,\theta_{n3,K_{n}}\right]}^{T}$, $\theta_{n4}={\left[\theta_{n4,1},\cdots ,\theta_{n4,K_{n}}\right]}^{T}$, $\psi_{n}={\left[\varphi_{n,1},\cdots ,\varphi_{n,K_{n}}\right]}^{T}$, $\theta_{c1}={\left[\theta_{c1,K_{n}+1},\cdots ,\theta_{c1,K}\right]}^{T}$, $\theta_{c2}={\left[\theta_{c2,K_{n}+1},\cdots ,\theta_{c2,K}\right]}^{T}$, $\theta_{c3}={\left[\theta_{c3,K_{n}+1},\cdots ,\theta_{c3,K}\right]}^{T}$, and $\theta_{c4}={\left[\theta_{c4,K_{n}+1},\cdots ,\theta_{c4,K}\right]}^{T}$. Then, followed by references [40,41,42,43], the *(p, q)*th entry of the $\left(5K_{n}+4K_{c})\times (5K_{n}+4K_{c}\right)$ CRB matrix for the parameter ω estimates is given by

(32) [CRB−1(ω)]p,q=2Lσn2Re{tr[∂A∂ωpPA⊥∂A∂ωqQ]}

where $\omega_{k}$ is the *k*th element of ω,

(33) $$A=\left[\begin{array}{cc} C_{n}\Phi_{1} & C_{c} \end{array}\right]$$

(34) $$P_{A}^{⊥}=I-A{\left(A^{H}A\right)}^{-1}A^{H}$$

(35) $$Q=P_{s}A^{H}R^{-1}AP_{s}=\left[\begin{array}{cc} Q_{1} & Q_{2}\\ Q_{3} & Q_{4} \end{array}\right]$$

and $Q_{1}$, $Q_{2}$, $Q_{3}$, and $Q_{4}$ are sizes of $K_{n}\times K_{n}$, $K_{n}\times K_{c}$, $K_{c}\times K_{n}$, and $K_{c}\times K_{c}$ matrices, respectively,

(36) $$P_{s}=E(\left[\begin{array}{c} s_{n}\\ s_{c} \end{array}\right]{\left[\begin{array}{c} s_{n}\\ s_{c} \end{array}\right]}^{H})$$

(37) $$R=E[xx^{H}]$$

Define

(38) $$D_{n}=\left[D_{\theta_{n1}},D_{\theta_{n2}},D_{\theta_{n3}},D_{\theta_{n4}},\frac{j}{2}C_{n}\Phi_{1},0_{MN\times 4K_{c}}\right]$$

(39) $$D_{c}=\left[0_{MN\times 5K_{n}},D_{\theta_{c1}},D_{\theta_{c2}},D_{\theta_{c3}},D_{\theta_{c4}}\right]$$

with $D_{\theta_{n1}}=\left[\frac{\partial C_{n}}{\partial \theta_{n1,1}}\Phi_{1},\ldots ,\frac{\partial C_{n}}{\partial \theta_{n1,K_{n}}}\Phi_{1}\right]$, $D_{\theta_{n2}}=\left[\frac{\partial C_{n}}{\partial \theta_{n2,1}}\Phi_{1},\ldots ,\frac{\partial C_{n}}{\partial \theta_{n2,K_{n}}}\Phi_{1}\right]$, $D_{\theta 3}=\left[\frac{\partial C_{n}}{\partial \theta_{n3,1}}\Phi_{1},\ldots ,\frac{\partial C_{n}}{\partial \theta_{n3,K_{n}}}\Phi_{1}\right]$, $D_{\theta_{n4}}=\left[\frac{\partial C_{n}}{\partial \theta_{n4,1}}\Phi_{1},\ldots ,\frac{\partial C_{n}}{\partial \theta_{n4,K_{n}}}\Phi_{1}\right]$, $D_{\theta_{c1}}=\left[\frac{\partial C_{c}}{\partial \theta_{c1,K_{n}+1}},\ldots ,\frac{\partial C_{c}}{\partial \theta_{c1,K}}\right]$, $D_{\theta_{c2}}=\left[\frac{\partial C_{c}}{\partial \theta_{c2,K_{n}+1}},\ldots ,\frac{\partial C_{c}}{\partial \theta_{c2,K}}\right]$, $D_{\theta_{c3}}=\left[\frac{\partial C_{c}}{\partial \theta_{c3,K_{n}+1}},\ldots ,\frac{\partial C_{c}}{\partial \theta_{c3,K}}\right]$, and $D_{\theta_{c4}}=\left[\frac{\partial C_{c}}{\partial \theta_{c4,K_{n}+1}},\ldots ,\frac{\partial C_{c}}{\partial \theta_{c4,K}}\right]$.

After some simplifications, we obtain the closed-form expression for the CRB ω as

(40) $$CRB(\omega )=\frac{\sigma_{n}^{2}}{2L}{\left[\begin{array}{cc} CRB_{1}(\omega ) & CRB_{2}(\omega )\\ CRB_{3}(\omega ) & CRB_{4}(\omega ) \end{array}\right]}^{-1}$$

where

(41) CRB1(ω)=Re{Jn(DnHPA⊥Dn)JnT⊙(15⊗15T⊗Q1T)}−1

(42) CRB2(ω)=Re{Jn(DnHPA⊥Dc)JcT⊙(14⊗15T⊗Q3T)}−1

(43) CRB3(ω)=Re{Jc(DcHPA⊥Dn)JnT⊙(15⊗14T⊗Q2T)}−1

(44) CRB4(ω)=Re{Jc(DcHPA⊥Dc)JcT⊙(14⊗14T⊗Q4T)}−1

with $J_{n}=\left[\begin{array}{cc} I_{5K_{n}} & 0_{5K_{n}\times 4K_{c}} \end{array}\right]$, $J_{c}=\left[\begin{array}{cc} 0_{4K_{c}\times 5K_{n}} & I_{4K_{c}} \end{array}\right]$, $1_{5}=\left[1,1,1,1,1\right]$ and $1_{4}=\left[1,1,1,1\right]$.

It should be noted that the CRB(ω) will degenerate into $CRB_{1}(\omega )$ and $CRB_{4}(\omega )$, which corresponds to the incident sources are all strictly noncircular and circualr sources, respectively, namely, $CRB(\omega )=CRB_{1}(\omega )$ and $CRB(\omega )=CRB_{4}(\omega )$.

## 5. Simulation Results

In this part, we evaluate the effectiveness of the proposed method in terms of several simulations. The proposed algorithm is compared with Xia’s algorithm [17], asymptotic performance analysis (Proposed asy.) in Equations (30) and (31), and the derived stochastic CRB in Equation (40). We use the root mean square error (RMSE) given by $RMSE\_DOD=\sqrt{\frac{1}{KM_{c}}\sum_{k=1}^{K}\sum_{m=1}^{M_{c}}\left[{\left({\hat{\theta }}_{k1}-\theta_{k1}\right)}^{2}+{\left({\hat{\theta }}_{k2}-\theta_{k2}\right)}^{2}\right]}$ and $RMSE\_DOA=\sqrt{\frac{1}{KM_{c}}\sum_{k=1}^{K}\sum_{m=1}^{M_{c}}\left[{\left({\hat{\theta }}_{k3}-\theta_{k3}\right)}^{2}+{\left({\hat{\theta }}_{k4}-\theta_{k4}\right)}^{2}\right]}$ as the performance criterion, where $M_{c}$ is the number of Monte-Carlo trials. The first experiment is based on an L-shaped MIMO array with $N_{1}=3,M_{1}=M_{2}=N_{2}=2$, and for the next two experiments, $M_{1}=M_{2}=N_{1}=N_{2}=3$, d is half wavelength. 

**Figure 4:**
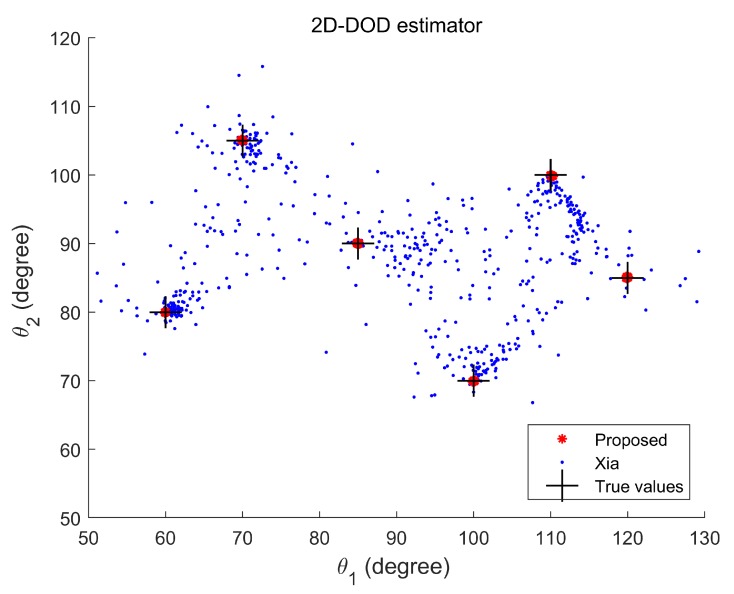
Two dimensional (2D)-direction of departure (DOD) scattergram of six targets for mixed signals.

**Figure 5:**
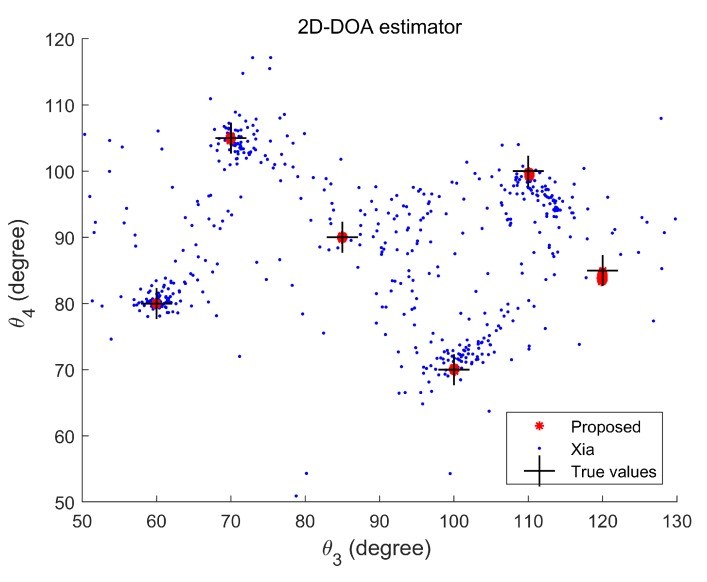
2D-DOA scattergram of six mixed signals.

**Experiment 1.** In the first experiment, we verify that the mixture of strictly noncircular and circular signals can be estimated successfully by the proposed method with the increasing of the maximum number of detectable signal in comparison with Xia’s method. Here, we consider six uncorrelated targets in the experiment, which have five BPSK signals with direction pairs $\left(70^{{}^{\circ}},105^{{}^{\circ}},70^{{}^{\circ}},105^{{}^{\circ}}\right)$, $\left(60^{{}^{\circ}},80^{{}^{\circ}},60^{{}^{\circ}},80^{{}^{\circ}}\right)$, $\left(85^{{}^{\circ}},90^{{}^{\circ}},85^{{}^{\circ}},90^{{}^{\circ}}\right)$, $\left(100^{{}^{\circ}},70^{{}^{\circ}},100^{{}^{\circ}},70^{{}^{\circ}}\right)$, and $\left(110^{{}^{\circ}},100^{{}^{\circ}},110^{{}^{\circ}},100^{{}^{\circ}}\right)$, and one QPSK signal with direction pairs $\left(120^{{}^{\circ}},85^{{}^{\circ}},120^{{}^{\circ}},85^{{}^{\circ}}\right)$. Then, we have $K_{n}+2K_{c}=7$, $\min{\left\{2N(M_{1}-1),2N(M_{2}-1)\right\}}_{DOD}=8$, and $\min{\left\{2M(N_{1}-1),2M(N_{2}-1)\right\}}_{DOA}=6$. The signal-to-noise ratio (SNR) is set at 25 dB, the number of snapshots is 500, and $M_{c}=100$. Figure 4 and Figure 5 show the 2D-DODs and 2D-DOAs scattergram of six mixed signals, respectively. It can be seen that the proposed algorithm can estimate the 2D-DODs and 2D-DOAs of six targets correctly, while the algorithm in reference [17] fails to work, because the former can detect more signals with available noncircular information. It should also be noted that the 2D-DOA estimation for QPSK signal is slightly inaccurate, because the number of mixed targets has exceeded the maximum number that the 2D-DOA can detect, but it still can roughly estimate the 2D-DOA of QPSK signal. 

**Figure 6:**
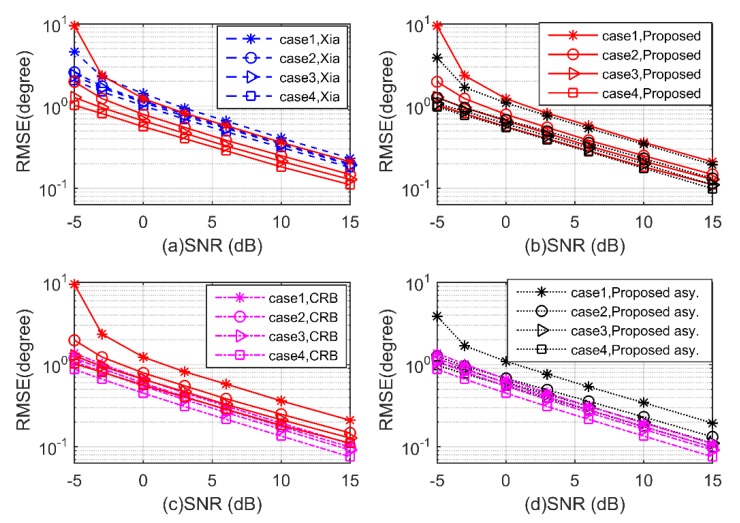
Root mean square error (RMSE) of 2D-DOD for mixed signals versus signal-to-noise ratio (SNR) (**a–d**).

**Figure 7:**
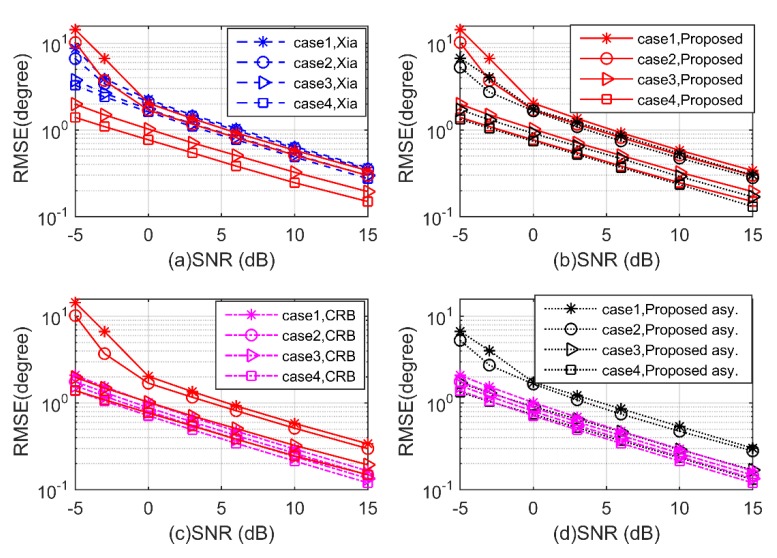
RMSE of 2D-DOA for mixed signals versus SNR (**a–d**).

**Experiment 2.** In the second experiment, the performance of the proposed algorithm is investigated with SNR varying from −5 dB to 15 dB. We consider four uncorrelated mixed signals with direction pairs $\left(60^{{}^{\circ}},50^{{}^{\circ}},60^{{}^{\circ}},50^{{}^{\circ}}\right)$, $\left(70^{{}^{\circ}},50^{{}^{\circ}},80^{{}^{\circ}},70^{{}^{\circ}}\right)$, $\left(70^{{}^{\circ}},60^{{}^{\circ}},80^{{}^{\circ}},90^{{}^{\circ}}\right)$, and $\left(80^{{}^{\circ}},70^{{}^{\circ}},100^{{}^{\circ}},90^{{}^{\circ}}\right)$. We consider the cases of one, two, three, and four BPSK signals, and the remaining signals are QPSK, respectively. The number of snapshots is 300 and $M_{c}$ = 2000. In Figure 6 and Figure 7, the estimation performance of the proposed algorithm is shown to be superior to Xia’s algorithm for both 2D-DOD and 2D-DOA estimation in all four cases. From case 1 to case 4, the performance of the proposed algorithm is improved in turn, as more noncircular information is available. In addition, the RMSEs of the proposed algorithm vary almost in accordance with their asymptotic error-variances, and both of them are close to the CRBs, especially for case 3 and case 4.

**Figure 8:**
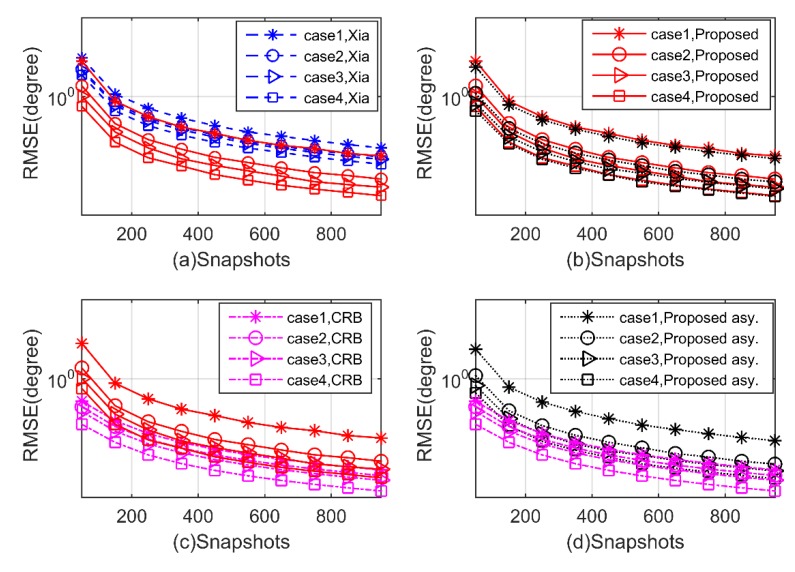
RMSE of 2D-DOD for mixed signals versus snapshots (**a–d**).

**Figure 9:**
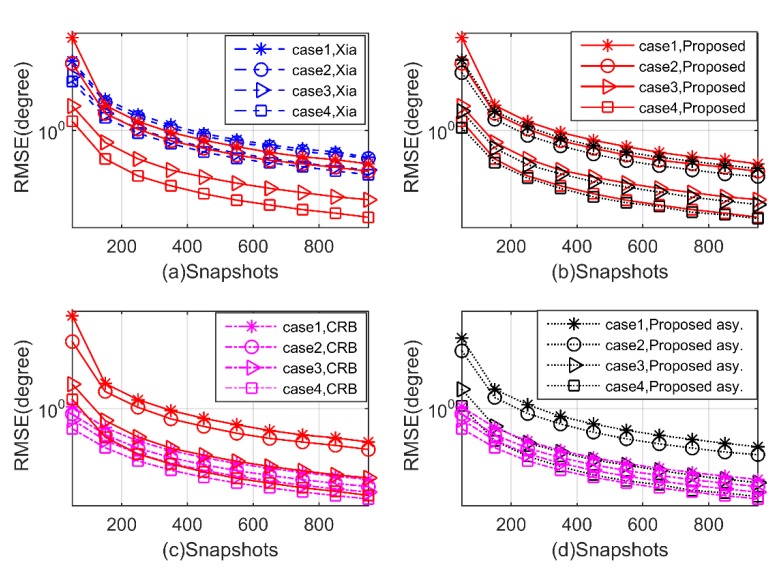
RMSE of 2D-DOA for mixed signals versus snapshots (**a–d**).

**Experiment 3.** In the third experiment, we investigate the performance with respect to a varying number of snapshots ranging from 50 to 950. The SNR is set at 5Db, and the other parameters are the same as Experiment 2. As shown in Figure 8 and Figure 9, we can draw similar conclusions as Experiment 2, that the proposed algorithm has better performance with the number of snapshots increases in all four cases, again outperforms the Xia’s method, and is close to the theoretical benchmark.

## 6. Conclusions

Based on the joint diagonalization technique, a 2D-DOD and 2D-DOA estimation algorithm for mixed strictly noncircular and circular signals in L-shaped bistatic MIMO radar is proposed in this paper. It utilizes the noncircularity characteristic to construct a virtual array, and then derives the joint diagonalization-based NC-ESPRIT method to achieve automatic pairing and the identification of the estimated 4D angles of mixed signals. The asymptotic performance of the proposed method as well as the stochastic CRB for the mixed signals scenario is also derived. Simulation results show that the proposed algorithm has a better angle estimation performance than the algorithm without noncircularity characteristics. 

## Figures and Tables

**Table 1:** Summary of the proposed method.

Input: $\;{\left\{\hat{x}(t)\right\}}_{t=1,\ldots T}$ : *T* snapshots of the new constructed array vector. Output: $\;{\left\{{\hat{\theta }}_{kl}\right\}}_{k=1,\ldots K,l=1,2,3,4}$: pair-free 2D-DODs and 2D-DOAs of *K* mixed signals
Step 1: Perform SVD on $\hat{Y}$ to get ${\hat{U}}_{s}$, and then compute ${\hat{E}}_{s}={\hat{U}}_{s}{\hat{\Sigma }}_{s}$; Step 2: Define a set $\hat{G}=\left\{{\hat{G}}_{1},{\hat{G}}_{2},{\hat{G}}_{3},{\hat{G}}_{4}\right\}$ according to Equation (18) Step 3: Implement the joint diagonalization to the set $\hat{G}$ to obtain the unitary matrix $\hat{E}$ by a series of Givens rotations; Step 4: Compute the eigenvalues ${\hat{\eta }}_{kl}$ according to Equation (20), and then compute ${\hat{\theta }}_{kl}$ according to Equation (21); Step 5: Compute the 2-D DODs and 2-D DOAs of circular signals according to Equation (22).

**Table 2:** Maximum number of detection signals.

Algorithm	Angle	Maximum Number
Proposed algorithm	DOD	$K_{n}+2K_{c}=\min\{2N(M_{1}-1),2N(M_{2}-1)\}$
	DOA	$K_{n}+2K_{c}=\min\{2M(N_{1}-1),2M(N_{2}-1)\}$
Xia’s algorithm	DOD	$K_{n}+K_{c}=\min\{N(M_{1}-1),N(M_{2}-1)\}$
	DOA	$K_{n}+K_{c}=\min\{M(N_{1}-1),M(N_{2}-1)\}$
